# Supplementary material for: Molecular and cellular determinants of motor asymmetry in zebrafish
Source: Nat Commun. 2020 Mar 3;11:1170. doi: 10.1038/s41467-020-14965-y (PMC7054361; doi:10.1038/s41467-020-14965-y)
Supplement: Supplementary file 1 — Supplementary Information [file 41467_2020_14965_MOESM1_ESM.pdf]

Supplementary information for
Horstick et al, Molecular and Cellular Determinants of Motor Assymetry in Zebrafish

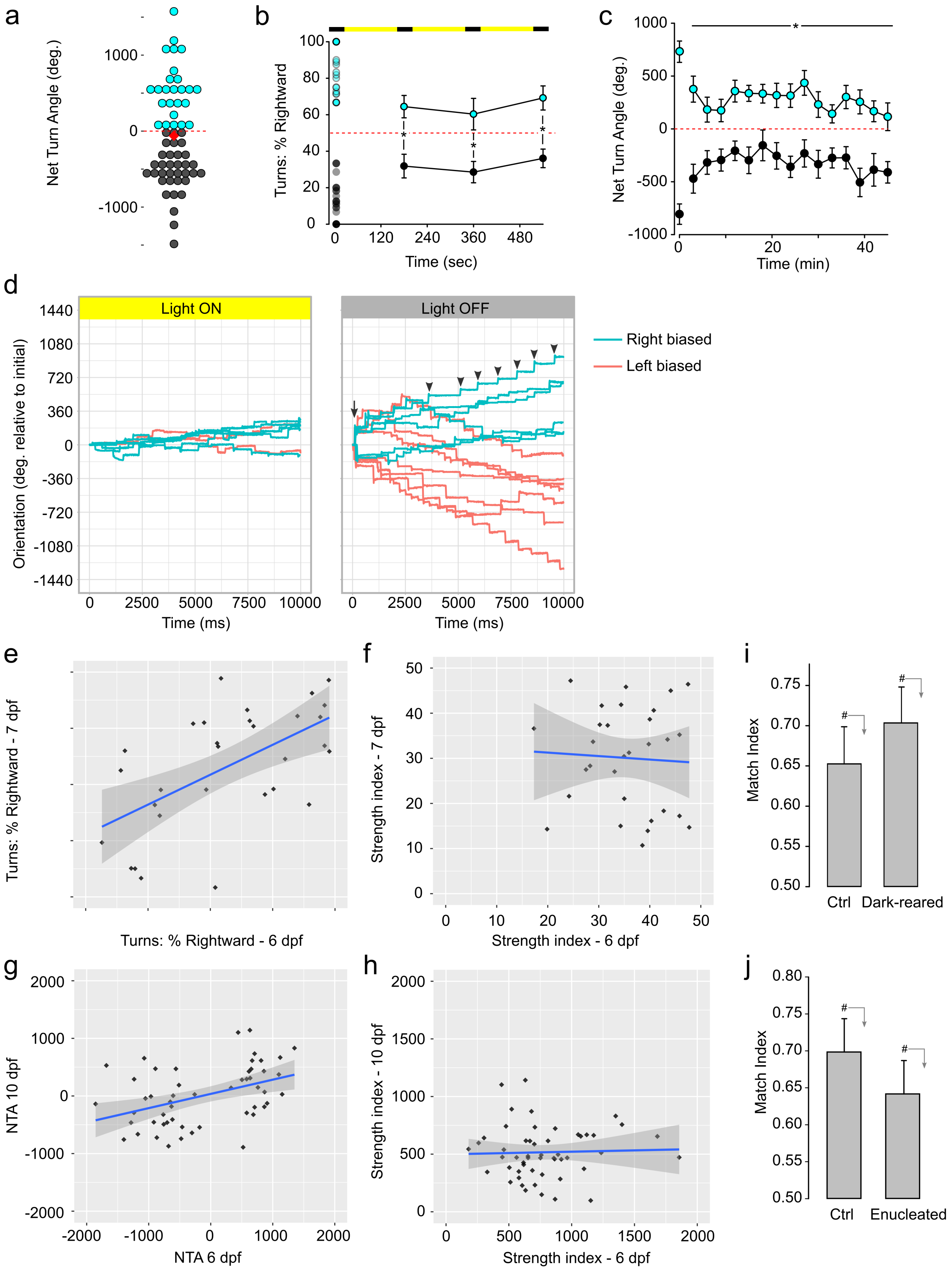

Supplementary Figure 1

Supplementary Figure 1. Zebrafish larvae exhibit left/right motor bias.

a. Dotplot of NTA during 30 s after loss of illumination for larvae in Figure 1B. Right-bias (cyan, N=25). Left-bias (Grey, N=34). Diamond: population mean.

b. Percentage of routine turns made in a rightward direction. Larvae were classified based on the first trial (dotplot at time 0): those with less than 33% of turns in a rightward direction were classified as left-biased (grey, N=24), and those with greater than 66% as right-biased (cyan, N=22). Trials 2-4 show means for each group. Repeated measures ANOVA effect of first-trial direction $F_{1,44}$=25.3, $\eta^2_p = 0.37$, $p < 0.001$. Asterisk $p < 0.05$ t-test.

c. NTA for 16 dark trials for larvae classified based on first trial responses as right (cyan, N=17) or left (grey, N=24) biased. Each dark trial lasted 30 s, with 180 s of illumination between trials. Repeated measures ANOVA effect of first-trial direction $F_{1,30}$=21.0, $\eta^2_p = 0.41$, $p < 0.001$ Asterisk $p < 0.05$ between groups at all timepoints, t-test.

d. Orientation of larvae relative to initial orientation every 10 ms over a 10 s period following loss of illumination (right panel, N=13 larvae) or during constant illumination (left panel, N=8). Larvae were classified as left (red) or right-biased (blue) based on the mean percent of turns in a rightward direction over four trials, and traces show the first of the four trials. Large changes in orientation at the start of the light-OFF condition are due to O-bend responses (arrow), whereas small subsequent changes are routine turns (arrowhead)

e. Percentage of turns made in a rightward direction (mean of 4 trials) for larvae tested at 6 dpf and again at 7 dpf (N=30).

f. Strength index of routine-turn direction bias for larvae in (d). Index is calculated by taking the absolute difference between the percentage of routine turns executed in a rightward direction and 50% (e.g. 30% rightward turns would give a value of 20 ; 100% would give 50) for each of the four trials per larva, then taking the mean of the four values. This index measures the 'commitment' of a larvae to circle in a given direction on each day.

g. NTA (mean of four trials) for larvae tested at 6 dpf and 10 dpf (N=54).

h. Strength index of direction bias for larvae tested in (f). Index is the mean of the absolute values of the NTA for each of the four trials per larva

i. Match index for control (N=47) and dark-reared (N=41) larvae. # $p < 0.05$, $r = 0.58$, 0.58 respectively, one-sample permutation analysis to 0.5

j. Match index for control (N=42) and dual enucleated (N=40) larvae following the loss of illumination. # $p < 0.05$, $r = 0.64$, 0.32 respectively, one-sample permutation analysis to 0.5. Error bars represent standard error of the mean.
Source data are provided as a Source Data file.

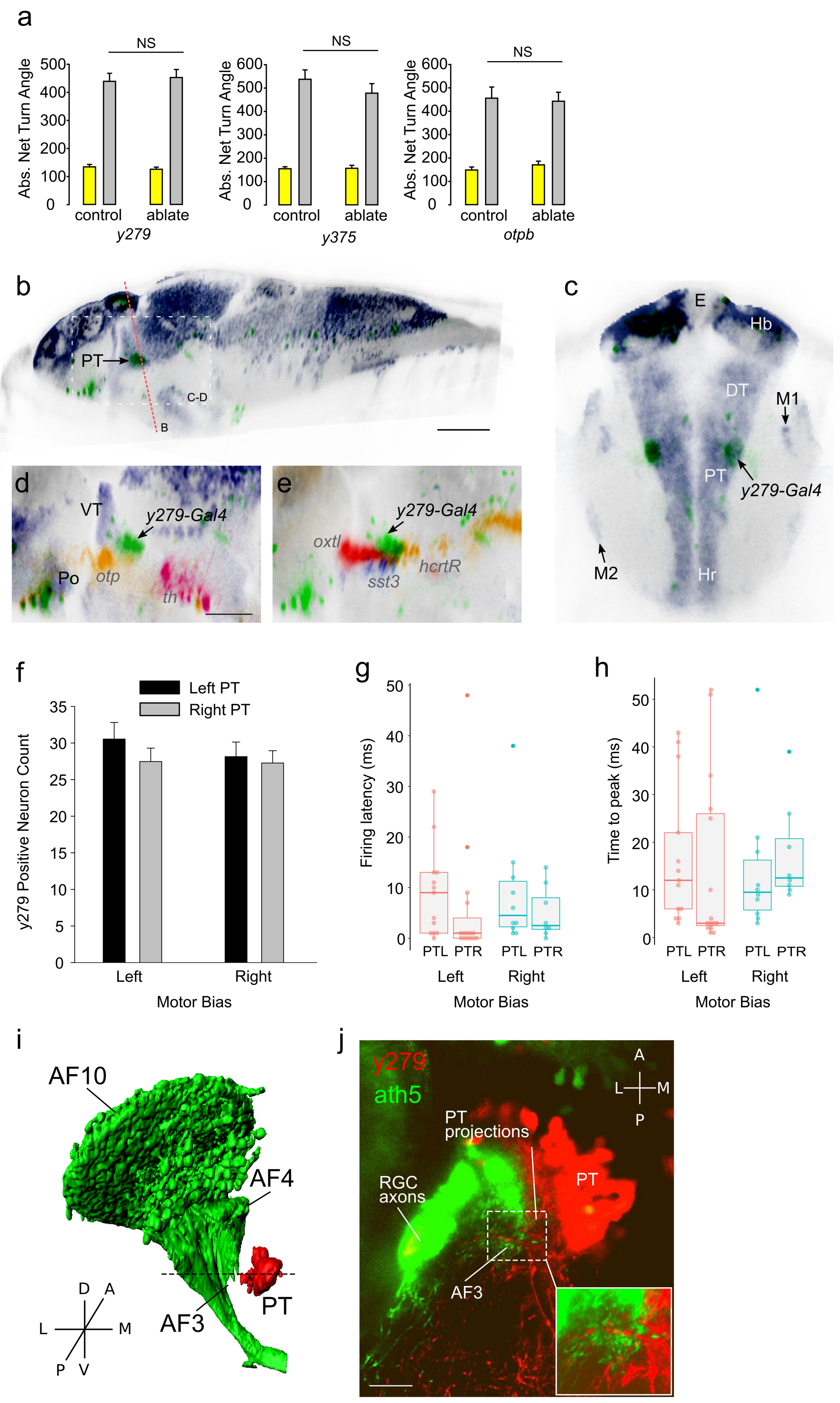

Supplementary Figure 2

Supplementary Figure 2. ***y279-Gal4*** **expressing neurons in the medial diencephalon are part of the posterior tuberculum.**

a. Total turning for drug treated controls (*y279,* N=54 ; *y375,* N=43 ; *otpbA,* N=35) and following chemogenetic ablation (*y279* N=58 ; *y375* N=37 ; *otpbA* N=46) in baseline (yellow) and dark (grey).

b-e. The medial diencephalic cluster in *y279-Gal4* is within the posterior tuberculum by comparison to the Mueller and Wullimann atlas and Z-Brain[1,2]. In the online version of ZBB (zbbrowser.com) these neurons are located at coordinates 272,307,209 (sagittal, transverse, horizontal planes). ZBB sagittal (b) and transverse (c) sections (sagittal 272 ; transverse 270 with oblique angle of 12 deg) displaying *huC:nls-mCar* (purple) and *y279-Gal4* (green) for comparison to the Mueller and Wullimann atlas Hu-protein antistain in 5 dpf brain panel 31 (page 125). *y279* neurons are within the central hu+ domain, between M1 and M2 migrated cell groups, annotated as the ventral posterior tuberculum. PT neurons project to the habenula, although in a previous report, habenula-projecting PT neurons were assigned to M2 which is lateral to the *y279* cluster 51. Scale bar 100 µm. d-e. Same ZBB sagittal section with (d) *y279-Gal4* (green), *th:Gal4* (pink), *otpB.A:Gal4* (orange) and *gad1b:GFP* (blue). (e) *y279-Gal4* (green), *sst3:Gal4* (blue), *hcrtR:Gal4* (orange) and *oxtl:GFP* (blue).
Mid-diencephalic *y279* neurons do not overlap with GABA transgenic marker gad1b:GFP, and are therefore unlikely to be part of the ventral thalamus[3]. The *y279* cluster is within the PT mask in Z-Brain, although a small section at the dorsal aspect enters the neighboring preoptic area[2]. *y279* neurons are a non-dopaminergic rostral lobe derived from the embryonic ventral PT. Developmentally, the PT originates from the basal plate of prosomeres 1-3 and ventral PT specifically from prosomere 3[4]. Whereas teleost PT DA neurons and mammalian DA neurons of the substantia nigra/ventral tegmental area are likely homologous, mammalian non-DA derivatives of basal prosomeres are less clear: the basal plate of prosomere 3 gives rise to the Fields of Forel[5]. Scale bar 50 µm. DT, thalamus. E, epiphysis. Hb, habenula. Hr, rostral hypothalamus. M1, migrated pretectal area. M2, migrated posterior tubercular area. Po, preoptic region. PT, posterior tuberculum. VT, ventral thalamus.

f. *y279-Gal4* expressing rostral PT neuron counts (black, left hemisphere ; grey, right hemisphere) in left (N=13) and right (N=15) biased larvae.

g-h. Latency to firing (g) and time from light-OFF to peak neuron activity (h) for neurons that respond to light OFF in left (PTL) and right (PTR) PT, for larvae classified as left (red) or right (blue) motor biased. No significant effect of interaction between PT hemisphere and motor-bias: $F_{1,42}$=.016, p =0.9 and $F_{1,42}$=.34, p =0.56 respectively.

i. 3D rendering showing proximity of a RGC projections (*atoh7:GFP,* green) to rostral PT neurons in *y279-Gal4, UAS:Kaede* embryos (photoconverted kaede; red). Salient RGC AFs are indicated.

j. Confocal plane at the level of dotted black line in (g). AF3 and rostral PT projections are indicated. Inset: magnified view of boxed area showing proximity of projections from RGCs and rostral PT neurons. Scale bar 20µm. Error bars, standard error of the mean. Box plots, median and quartiles with whiskers indicating 10-90%.
Source data are provided as a Source Data file.

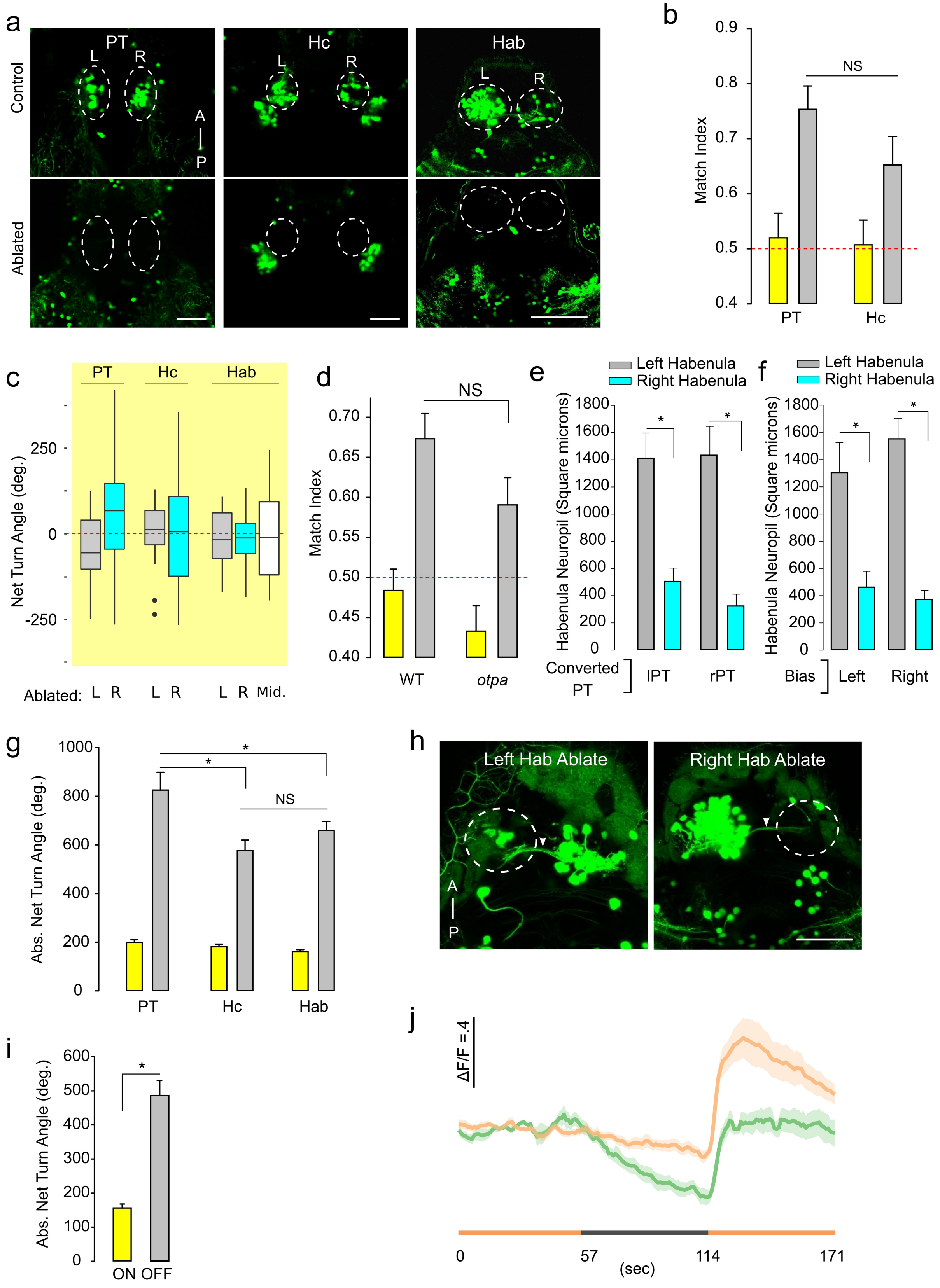

Supplementary Figure 3

Supplementary Figure 3. Behavioral analysis after posterior tuberculum and habenula ablations.

a. Confocal projection of *y279* expressing posterior tuberculum (PT), caudal hypothalamus (Hc) and habenula (Hab) neurons in representative control (top) and laser ablated (bottom) larvae. Scale bar 50 µm.

b. Match index after unilateral ablations of *y279* posterior tuberculum (PT, N=50) or caudal hypothalamus neurons (Hc, N=46) during baseline illumination (yellow) or dark-induced circling (grey). Left and right hemisphere ablations are combined in this analysis.

c. Net turn angle (mean of trials 1-4) under baseline illumination after unilateral ablation of left (grey, PT N=27 ; Hc N=24 ; Hab N=33) or right (cyan, PT N=23 ; Hc N=22 ; Hab N=28) hemisphere *y279* PT or Hc neurons or section of the habenula commissure (white, N=24).

d. Match index for cousin wildtype (N=103) and *otpa* mutant larvae (N=92). Dotted red line denotes random motor bias.

e-f. Neuropil area in the left and right habenulae following unilateral photoconversion of the left or right PT (e), in larvae (N=27) previously sorted as left or right motor-biased (f). Three-way ANOVA for habenula area by PT-side photoconverted, motor-bias and habenula-hemisphere revealed only a strong main effect for the habenula hemisphere ($F_{[1,26]}=41.2$, $p< 0.001$, d=2.3) and no other main effects or interactions. ANOVA: main effect for habenula hemisphere $F_{1,26}=41.2$, $\eta^2_p = 0.61$, $p < 0.001$.

g. Total turning after unilateral laser ablations of *y279* posterior tuberculum (PT, N=50), caudal hypothalamus (Hc, N=46) or habenula (Hab, N=61) neurons under baseline illumination (yellow) and during dark trials (grey). Left and right hemisphere ablations are combined in this analysis. Asterisk $p < 0.05$, $d = 0.59$, 0.41 respectively, t-test.

h. Confocal projections following unilateral ablation of the *y279-Gal4* expressing neurons in the left or right habenula hemispheres. Arrowheads indicate labeled fibers within the habenular commissure. Scale bar 50 µm.

i. Total turning behavior following habenula commissure section for larvae in Figure 5g. Asterisk $p < 0.05$, $d = 2.1$, t-test.

j. Mean and standard error for GCaMP fluorescence in neurons with decreased activity to light OFF (green, N=5) and neurons that responded to light ON (orange, N=9). An additional 2 neurons (one in the left hemisphere, and one on the right out of 101 neurons recorded), showed increased activity at the light OFF transition. Error bars represent standard error of the mean. Box plots show median and quartiles with whiskers indicating 10-90%.
Source data are provided as a Source Data file.

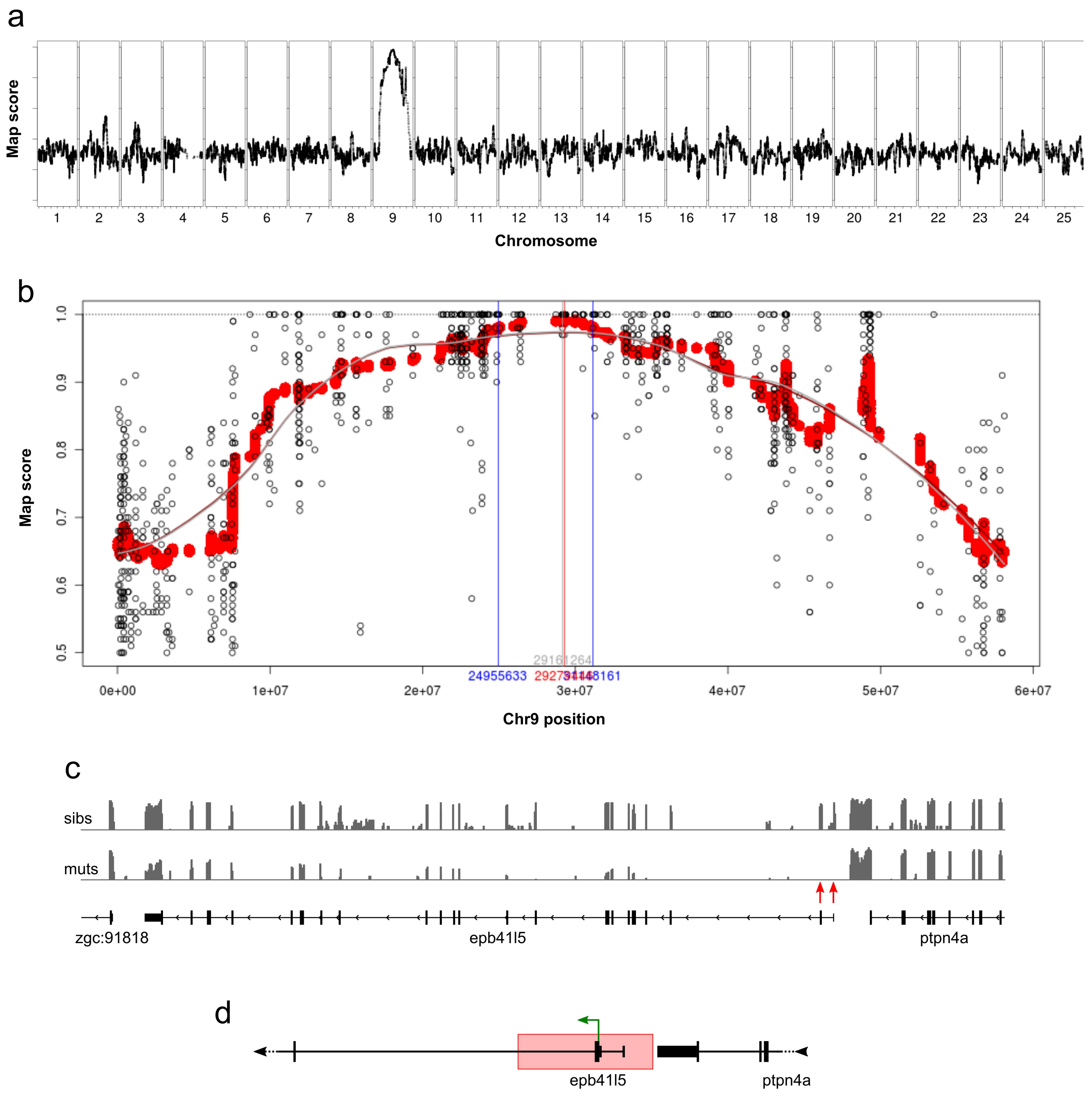

Supplementary Figure 4

Supplementary Figure 4. Mapping of the *moe*^{y606} mutation.

a. Whole genome RNAmapper pipeline map scores, comparing pooled mutants with the curly-up phenotype and siblings

b. Map scores for chromosome 9.

c. Read counts (IGVtools count function) from bulk RNAseq for curly-up mutants and siblings. Arrows indicate a complete lack of reads for exons 1 and 2 of *epb41l5* (one read mapped to exon 3).

d. Schematic of the deletion in *moe*^{y606} which eliminates the first two exons of *epb41l5*. The deletion removes bases 213682-218117 from chr9_KZ114909v1_alt (an alternative build for part of chromosome 9) and inserts 53 low complexity bases that are not found in this region.

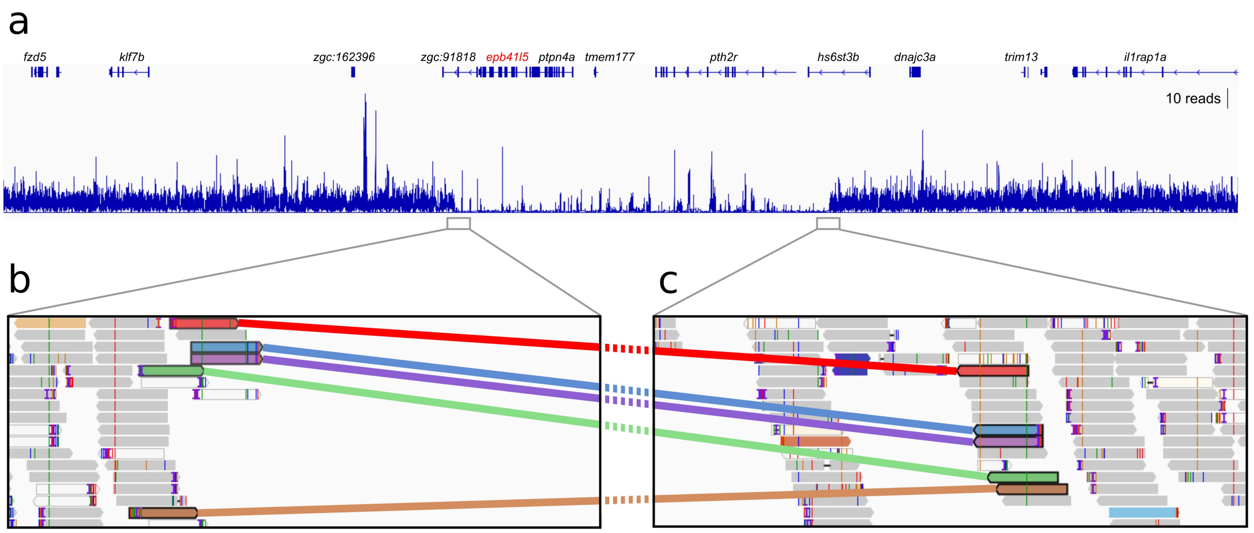

Supplementary Figure 5

Supplementary Figure 5. Mapping of the *moe*^{b476} deletion.

a. RNAseq read mapping from *moe*^{b476} mutant larvae, in the region chr9:28,190,541 to 29,960,696. Grey boxes indicate the areas highlighted in (B).

b-c. RNAseq reads mapped to the left side of the deletion (b, chr9:28,835,314 to 28,836,377) and right side of the deletion (c, chr9:29,370,348 to 29,371,411). The 6 colored reads indicate reads where one of the paired ends mapped to one side of the deletion, and the other paired end mapped to the other side of the deletion.

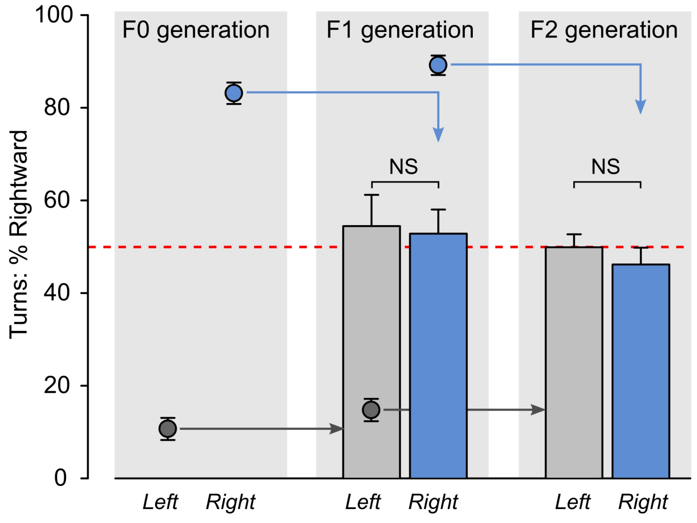

Supplementary Figure 6

Supplementary Figure 6. Left-right bias is not heritable.

Mean percent rightward turns for groups of larvae used to assess heritability of left/right motor asymmetry. F0 generation: circles indicate mean values of larvae selected to raise to become parents of the F1 generation (left, N=10 ; right, N=8). F1 generation: bars indicate the population means for F1 larvae derived from left-biased (grey, N=31) or right-biased (blue, N=37) F0 adults. Circles indicate mean values of larvae raised as parents of the F2 generation (left, N=7 ; right, N=9). F2 generation: bars indicate populations means for F2 larvae (left, N=86 ; right, N=69). Error bars represent standard error of the mean.
Source data are provided as a Source Data file.

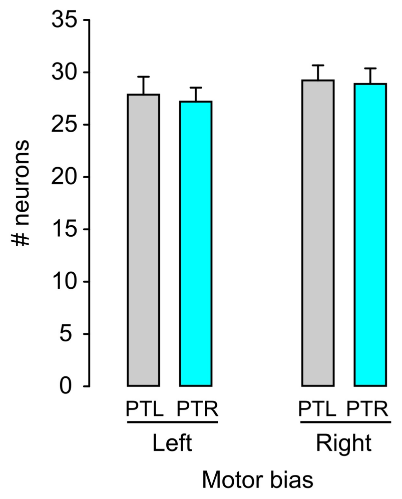

Supplementary Figure 7

Supplementary Figure 7. Neuron counts in left/right posterior tuberculum in heterozygous *moe*^{y606} larvae.

Number of *y279-Gal4, UAS:Kaede* expressing neurons in left (N=16) and right (N=10) biased *moe*$^{y606/+}$ larvae in the left PT (grey) and right PT (cyan). Error bars represent standard error of the mean. Source data are provided as a Source Data file.

Line	Neuroanatomical Regions
y249	olfactory sensory neurons, subpallium, pineal complex, Nuc MLF, posterior commissure, hypothalamus, retinotectal tract
y252	griseum tectale, optic tectum, hypothalamus, cerebellum, medulla, retina
y256	statoacoustic ganglion, retina
y275	olfactory sensory neurons, subpallium, preoptic region, pretectum, optic tectum, tegmentum, cerebellum, torus longitudinalis, hypothalamus, retina
y279	rostral posterior tuberculum, subpallium, preoptic, habenula (medial), hypothalamus, trigeminal ganglion, statoacoustic ganglion, optic tectum, retina
y294	pallium, subpallium, pineal complex, habenula, thalamus, posterior tuberculum, dorsal raphe, optic tectum, tegmentum, retina
y310	pineal complex, posterior tuberculum, hypothalamus
y313	posterior tuberculum, Nuc MLF, medulla, retina, spinal cord
y321	subpallium, preoptic, rostral hypothalamus, posterior tuberculum
y329	subpallium, ventral thalamus, posterior tuberculum, hypothalamus, optic tectum, motor neurons, retina
y355	dorsal raphe, medulla oblongata, retina, spinal cord
y375	rostral posterior tuberculum, subpallium, pineal complex, habenula (lateral), ventral thalamus, medulla oblongata, Nuc MLF, retina, spinal cord
y393	olfactory sensory neurons, pallium, subpallium, pineal, habenula (lateral), trigeminal ganglion, hypothalamus, pituitary, retina, spinal cord
y405	preoptic, trigeminal ganglion, locus coeruleus, optic tectum, medulla oblongata, retina, spinal cord
y425	olfactory bulb, hypothalamus, spinal cord
y441	pineal projection neurons, hypothalamus, vagal ganglion, retina, spinal cord
y467	subpallium, hypothalamus, tegmentum, dorsal raphe, Rhombomere 3, Rhombomere 5
y471	subpallium, preoptic, hypothalamus, oculomotor nucleus, interpeduncular nucleus tegmentum, dorsal raphe, medulla oblongata
y472	olfactory epithelium, preoptic, habenula, hypothalamus, tegmentum
y511	preoptic area, hypothalamus, medulla oblongata

Supplementary Table 1. Expression pattern of Gal4 in lines tested for motor-bias after ablation

Supplementary References

1. Mueller, T. & Wullimann, M. F. *Atlas of Early Zebrafish Brain Development. A Tool for Molecular Neurogenetics.* (Elsevier B.V., 2005).
2. Randlett, O. *et al.* Whole-brain activity mapping onto a zebrafish brain atlas. *Nat Methods* **12**, 1039–46 (2015).
3. Mueller, T. What is the Thalamus in Zebrafish? *Front Neurosci* **6**, 64 (2012). doi.org/10.3389/fnins.2012.00064
4. Vernier, P. & Wullimann, M. F. Evolution of the posterior tuberculum and preglomerular nuclear complex. *Encyclopedia of Neuroscience* 1404–1413 (2009).
5. Puelles, L. & Rubenstein, J. L. R. Forebrain gene expression domains and the evolving prosomeric model. *Trends in Neurosciences* **26**, 469–476 (2003).
