# Supplementary material for: Molecular and cellular determinants of motor asymmetry in zebrafish
Source: Nat Commun. 2020 Mar 3;11:1170. doi: 10.1038/s41467-020-14965-y (PMC7054361; doi:10.1038/s41467-020-14965-y)
Supplement: Supplementary file 3 — Reporting summary [file 41467_2020_14965_MOESM3_ESM.pdf]

nature research

Corresponding author(s): Eric J. Horstick and Harold A. Burgess (lead)

Last updated by author(s): Nov 12, 2019

Reporting Summary

Nature Research wishes to improve the reproducibility of the work that we publish. This form provides structure for consistency and transparency in reporting. For further information on Nature Research policies, see Authors & Referees and the Editorial Policy Checklist .

Statistics

For all statistical analyses, confirm that the following items are present in the figure legend, table legend, main text, or Methods section.

n/a	Confirmed	
☐	☒	The exact sample size (*n*) for each experimental group/condition, given as a discrete number and unit of measurement
☐	☒	A statement on whether measurements were taken from distinct samples or whether the same sample was measured repeatedly
☐	☒	The statistical test(s) used AND whether they are one- or two-sided<br>*Only common tests should be described solely by name; describe more complex techniques in the Methods section.*
☐	☒	A description of all covariates tested
☐	☒	A description of any assumptions or corrections, such as tests of normality and adjustment for multiple comparisons
☐	☒	A full description of the statistical parameters including central tendency (e.g. means) or other basic estimates (e.g. regression coefficient) AND variation (e.g. standard deviation) or associated estimates of uncertainty (e.g. confidence intervals)
☐	☒	For null hypothesis testing, the test statistic (e.g. *F*, *t*, *r*) with confidence intervals, effect sizes, degrees of freedom and *P* value noted<br>*Give P values as exact values whenever suitable.*
☐	☒	For Bayesian analysis, information on the choice of priors and Markov chain Monte Carlo settings
☒	☐	For hierarchical and complex designs, identification of the appropriate level for tests and full reporting of outcomes
☐	☒	Estimates of effect sizes (e.g. Cohen's *d*, Pearson's *r*), indicating how they were calculated

Our web collection on statistics for biologists contains articles on many of the points above.

Software and code

Policy information about availability of computer code

Data collection	Data was collected using custom software written in IDL. The following code availability statement was added to manuscript: The analysis codes that were used in this study are available from the corresponding author upon request.
Data analysis	Data analysis was performed using the following software: R studio 1.1.463, JASP 0.11.1, IDL 8.6.1 and Gnumeric 1.12.28. Custom analysis software was written in IDL. The following code availability statement was added to manuscript: The analysis codes that were used in this study are available from the corresponding author upon request.

For manuscripts utilizing custom algorithms or software that are central to the research but not yet described in published literature, software must be made available to editors/reviewers. We strongly encourage code deposition in a community repository (e.g. GitHub). See the Nature Research guidelines for submitting code & software for further information.

Data

Policy information about availability of data

All manuscripts must include a data availability statement. This statement should provide the following information, where applicable:

- Accession codes, unique identifiers, or web links for publicly available datasets
- A list of figures that have associated raw data
- A description of any restrictions on data availability

Gal4 lines are available from the Zebrafish International Resource Center (http://zebrafish.org). Further information and requests for image datasets and analysis software should be directed to Harold Burgess (burgessha@mail.nih.gov) or Eric Horstick (eric.horstick@mail.wvu.edu). Numerical data underlying Figs 1b-e, g-j, 2c-f, 3b, g-i, 4c-e, 5e-g, 6c-h and Supplementary Figs 1b,i-j, 2a, f, 3e-f, g, i-j, 6 and 7 are provided in a Source Data file.

Field-specific reporting

Please select the one below that is the best fit for your research. If you are not sure, read the appropriate sections before making your selection.

[x] Life sciences [ ] Behavioural & social sciences [ ] Ecological, evolutionary & environmental sciences

For a reference copy of the document with all sections, see nature.com/documents/nr-reporting-summary-flat.pdf

Life sciences study design

All studies must disclose on these points even when the disclosure is negative.

Sample size	In pilot studies we found that at least 10 wildtype individuals per left/right group were needed to statistically resolve left/right biased populations. We used this number as a guideline in testing ablated larvae, and also for testing the effects of gene mutations (where genotype groups could only be assigned through post-hoc genotyping).
Data exclusions	In Figure S1b, individuals were excluded with % Rightward Turns utilization between 33.3-66.7% on the first trial. The rationale was that throughout the manuscript, left/right classification of individuals was based on the first trial. However high speed recordings were limited to 10 s due to camera memory (compated to 30 s for low-speed recordings) and we therefore used only larvae that we could definitively classify based on trial 1.
Replication	In all behavioral experiments, N refers to the number of individual larvae. At least 3 biological replicates were performed for most experiments to ensure reproducibility. We also replicated findings using multiple experimental systems (low speed and high speed recordings). Note that for calcium imaging data the results of multiple biological replicates (larvae) are combined in Fig. 4.
Randomization	For experiments involving repeated testing of individual motor identity individuals were recorded in a randomly selected behavioral recording rig on successful trials limiting impact from a specific recording environment.
Blinding	For all mutant characterization, genotypes were determined via post-hoc genotyping. In experiments testing the response of identified left/right biased individuals we used ambiguous group labels during testing.

Reporting for specific materials, systems and methods

We require information from authors about some types of materials, experimental systems and methods used in many studies. Here, indicate whether each material, system or method listed is relevant to your study. If you are not sure if a list item applies to your research, read the appropriate section before selecting a response.

Materials & experimental systems

n/a	Involved in the study	
[x]	[ ]	Antibodies
[x]	[ ]	Eukaryotic cell lines
[x]	[ ]	Palaeontology
[ ]	[x]	Animals and other organisms
[x]	[ ]	Human research participants
[x]	[ ]	Clinical data

Methods

n/a	Involved in the study	
[x]	[ ]	ChIP-seq
[x]	[ ]	Flow cytometry
[x]	[ ]	MRI-based neuroimaging

Animals and other organisms

Policy information about studies involving animals; ARRIVE guidelines recommended for reporting animal research

Laboratory animals	Danio rerio, TL and WIK strains, 4-10 days post fertilization, sex indeterminate in larvae zebrafish
Wild animals	Study did not involve wild animals
Field-collected samples	Study did not involve field-collected samples
Ethics oversight	NICHD animal care and use committee

Note that full information on the approval of the study protocol must also be provided in the manuscript.
